# Post COVID‐19 condition: critical need for a clear definition and detailed pathophysiology

**DOI:** 10.1002/jcsm.13108

**Published:** 2022-10-17

**Authors:** Tom J. Kerkhoff, Braeden T. Charlton, Brent Appelman, Michele van Vugt, Rob C.I. Wüst

**Affiliations:** ^1^ Laboratory for Myology, Faculty of Behavioural and Movement Sciences, Amsterdam Movement Sciences Vrije Universiteit Amsterdam Amsterdam The Netherlands; ^2^ Center for Experimental and Molecular Medicine, Amsterdam UMC – location AMC Universiteit van Amsterdam Amsterdam The Netherlands; ^3^ Department of Internal Medicine, Division of Infectious Diseases, Amsterdam University Medical Centers ‐ Location AMC University of Amsterdam Amsterdam The Netherlands

Many people contracting a SARS‐CoV‐2 infection successfully recover within a few weeks. Despite this, COVID‐19‐like symptoms can persist in 10–60% of people.^1^, ^2^ Patients mainly suffer from dyspnoea, extreme fatigue, muscle pain, weakness and atrophy, orthostatic intolerance, concentration difficulties and post‐exertional malaise. Who is at risk of developing post COVID‐19 condition is currently unclear. Otherwise healthy people with a relatively mild SARS‐CoV‐2 infection or children do not seem to be protected against post COVID‐19 condition.^3^ Post COVID‐19 condition (also called long COVID or Post‐Acute Sequelae of SARS‐CoV‐2) is currently defined by the World Health Organization as ‘an illness that occurs in people who have a history of probable or confirmed SARS‐CoV‐2 infection; usually within three months from the onset of COVID‐19, with symptoms and effects that last for at least two months. The symptoms and effects of post COVID‐19 condition cannot be explained by an alternative diagnosis’.^4^ In its current definition, we believe that without critically evaluating alternative diagnoses, post COVID‐19 condition risks including different clinical entities which require a different treatment and diagnostic approach (*Figure* 1).

**Figure 1:**
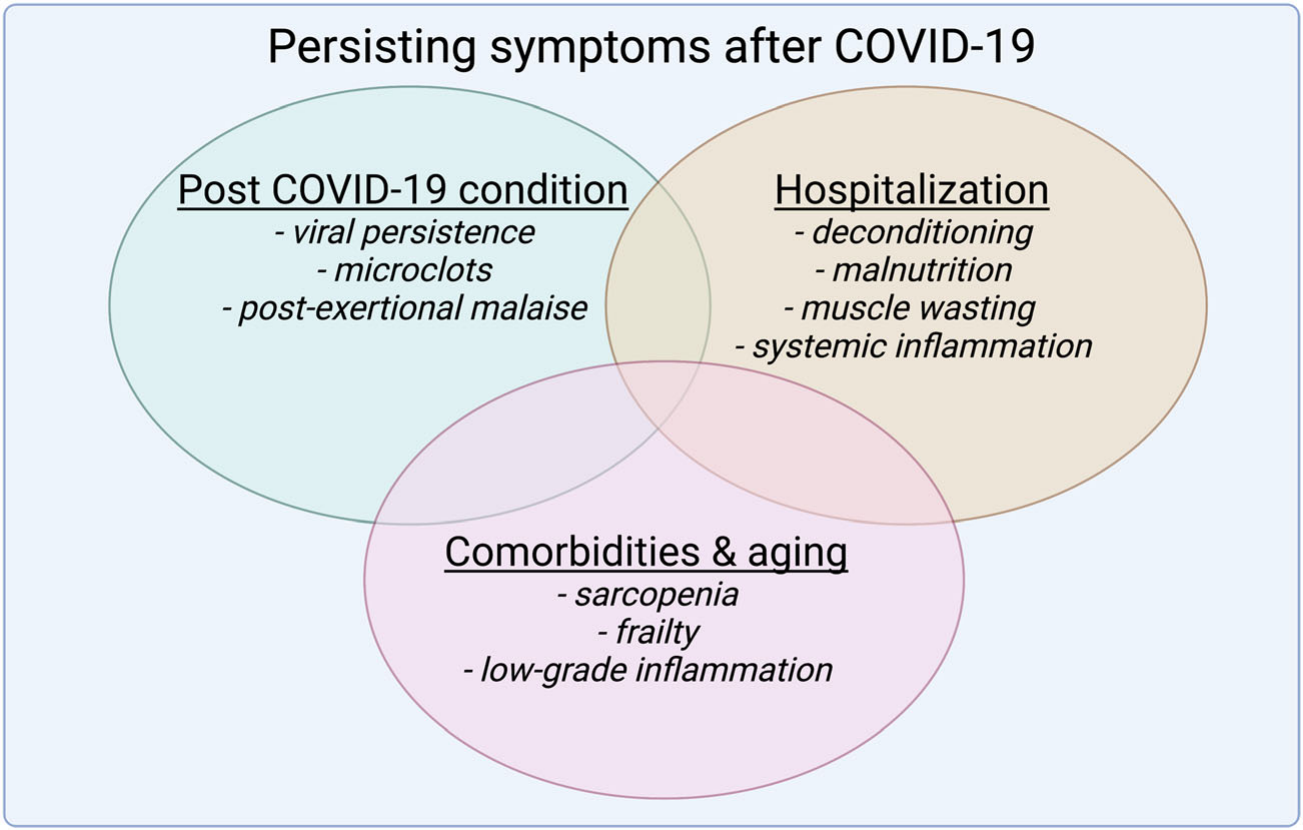
Multifactorial causes of persisting muscle weakness after COVID‐19. The current definition of post COVID‐19 condition of ‘having persisting symptoms after three months’ is too broad, as this includes patients who suffer from post‐intensive care syndrome and the associated slow recovery after hospitalization‐induced muscle weakness. Particularly, older people suffering from sarcopenia and patients with comorbidities generally exhibit muscle weakness and likely will have a reduced ability to fully recover from a SARS‐CoV‐2 infection. As such, a new definition of post COVID‐19 condition is required that exclude factors related to hospitalization, ageing and comorbidities.

Martone et al.^5^ recently reported exciting new clinical data in the *Journal of Cachexia, Sarcopenia and Muscle* on the prevalence of sarcopenia in the development of post COVID‐19 condition. They enrolled 541 recovered COVID‐19 patients with a variety of disease severity. Over 60% of patients were hospitalized, either with or without breathing support. A multidisciplinary clinical evaluation, physical performance assessment (consisting of a 6‐min walk test and a chair sit stand test) and a handgrip strength test were performed to assess the prevalence and severity of sarcopenia. About 20% of the participants were diagnosed with sarcopenia, and ~70% of sarcopenic patients were hospitalized during the acute infection, compared with ~60% in the non‐sarcopenic group. The time of hospitalization was longer in the sarcopenic group than the non‐sarcopenic group (27 ± 19 vs. 14 ± 10 days). The most prevalent comorbidities seen in patients with sarcopenia were inactivity, hypertension, diabetes and COPD. Severe COVID‐19 was more common in the sarcopenic group than in the non‐sarcopenic group. Dyspnoea and persisting fatigue after acute SARS‐CoV‐2 infection was higher in those diagnosed with sarcopenia. The authors conclude that sarcopenia is therefore a substrate for the development of post COVID‐19 condition. However, the heterogeneity in patient included in the study of Martone might cause difficulty in comparing the patients and understanding the pathophysiology of post COVID‐19 condition. Confounding factors may influence the relationship between sarcopenia and post COVID‐19 condition.

The average time between the acute SARS‐CoV‐2 infection and inclusion in the study of Martone et al.^5^ was 95 ± 51 days. The standard deviation indicates that a large proportion of patients were included within 3 months of initial infection. Whether these patients had sufficient time to recover from the hospitalization, and/or already were suffering from other comorbidities, such as age‐related sarcopenia, and/or had post COVID‐19 condition, however, remains unclear.

Independent of the underlying pathology, hospitalization itself can lead to a severe muscle loss in patients.^6^ Half the patients admitted to the intensive care unit are vulnerable to muscle wasting.^7^ This post‐intensive care syndrome or intensive care acquired weakness is a physical, cognitive and mental disorder that occurs during hospitalization or after hospital discharge^8^, ^9^ and contributes to the very long recovery process after hospital discharge. Symptoms include muscle weakness, exercise intolerance, fatigue and cognitive problems. As these symptoms overlap with the current diagnosis of post COVID‐19 condition, a portion of patients diagnosed with post COVID‐19 condition might (also) suffer from post‐intensive care syndrome, independent of the initial SARS‐CoV‐2 infection.^10^ Hospital duration and disease severity were indeed strong predictors of the development of sarcopenia in Martone et al.^5^ The underlying pathophysiology of post‐intensive care syndrome includes deconditioning‐induced muscle atrophy, which is worsened by malnutrition and systemic inflammation.^6^, ^10^, ^11^ Recovery after hospital discharge in older patients is generally long with a risk mortality and does not necessarily completely reverse the muscle mass loss.^12^

Older individuals and patients with chronic diseases, such as hypertension, diabetes, coronary artery disease and COPD, suffer from skeletal muscle weakness and have a higher prevalence of developing sarcopenia.^5^, ^13^, ^14^ Sarcopenia is characterized by the slow and progressive loss of muscle mass and strength, and is associated with ageing. Sarcopenia is an independent risk factor for frailty and mortality.^14^ Much is unknown about the speed of the progression of sarcopenia, but likely physical inactivity (COVID‐19‐pandemic‐related or otherwise) and comorbidities play a role in the progression of sarcopenia. Similarly, various chronic diseases are associated with a reduced muscle mass and weakness. As patients with chronic diseases are more likely to be hospitalized due to a more severe COVID‐19 phenotype, they will also suffer from more persisting symptoms after COVID‐19.^15^ Importantly, recovery from a viral infection or hospitalization is complicated by the observation of a slow recovery, or a blunted response to rehabilitation in older people and in patients with chronic diseases.^16^, ^17^ Older people and those with inflammation often do not fully recover within 3 months after hospitalization.^6^

Muscle weakness in patients recovering from a SARS‐CoV‐2 infection can therefore be multifactorial. Hospitalization, age and comorbidities are confounding factors in distinguishing what the underlying cause of muscle weakness is in patients who do not recover completely from COVID‐19.^10^ A clear diagnostic marker is currently missing to differentiate between patients with post COVID‐19 condition, post‐intensive care syndrome and those with an incomplete recovery after SARS‐CoV‐2 infection due to comorbidities and older age (*Figure* 1).

Recently, new additional diagnostic markers have been studied in the context of post COVID‐19 condition, in addition to symptom persistence after 3 months following initial SARS‐CoV‐2 infection. Microclots are one such proposed biomarker. Microclots containing amyloid fibrils have been found in the blood of patients with post COVID‐19 condition.^18^ It is, however, unclear whether microclots are exclusively found in patients with post COVID‐19 condition. With a better understanding of the pathophysiology of microclots, microclot testing in the blood may become an efficient diagnostic tool for patients with post COVID‐19 condition. Further, post‐exertional malaise seems unique to patients with post COVID‐19 condition and myalgic encephalomyelitis/chronic fatigue syndrome (ME/CFS). Post‐exertional malaise is the worsening of symptoms after performing exercise above an unknown threshold that include extreme fatigue, brain fog and fever.^19^ The observation that exercise worsens clinical symptoms in patients with post COVID‐19 condition, but not in other chronic diseases or after hospitalization, might provide new insights into a critically required new definition of post COVID‐19 condition.

The heterogeneity in patient inclusion poses a difficulty for clinicians treating patients and scientists studying the pathophysiology of post COVID‐19 condition. As such, clinicians and scientists are urged to critically evaluate confounding factors related to hospitalization, ageing and comorbidities into the diagnosis of post COVID‐19 condition. This will help in understanding post COVID‐19 condition and finding an effective treatment.

## Conflict of interest

The authors do not have any conflict of interest to declare.
